# Do attributes in the physical environment influence children's physical activity? A review of the literature

**DOI:** 10.1186/1479-5868-3-19

**Published:** 2006-07-27

**Authors:** Kirsten Krahnstoever Davison, Catherine T Lawson

**Affiliations:** 1 Department of Health Policy, Management and Behavior, University at Albany (SUNY), Albany, NY, USA; 2 Department of Geography and Planning, University at Albany (SUNY), Albany, NY, USA

## Abstract

**Background:**

Many youth today are physically inactive. Recent attention linking the physical or built environment to physical activity in adults suggests an investigation into the relationship between the built environment and physical activity in children could guide appropriate intervention strategies.

**Method:**

Thirty three quantitative studies that assessed associations between the physical environment (perceived or objectively measured) and physical activity among children (ages 3 to 18-years) and fulfilled selection criteria were reviewed. Findings were categorized and discussed according to three dimensions of the physical environment including recreational infrastructure, transport infrastructure, and local conditions.

**Results:**

Results across the various studies showed that children's participation in physical activity is positively associated with publicly provided recreational infrastructure (access to recreational facilities and schools) and transport infrastructure (presence of sidewalks and controlled intersections, access to destinations and public transportation). At the same time, transport infrastructure (number of roads to cross and traffic density/speed) and local conditions (crime, area deprivation) are negatively associated with children's participation in physical activity.

**Conclusion:**

Results highlight links between the physical environment and children's physical activity. Additional research using a transdisciplinary approach and assessing moderating and mediating variables is necessary to appropriately inform policy efforts.

## Background

Many youth today are physically inactive. Considerable evidence documents that nearly 35% of youth in the US fail to meet the minimum physical activity guidelines, and another 14% are completely inactive [1,2]. Low levels of physical activity and the failure to meet physical activity recommendations have notable health consequences among children including increased risk of obesity [3], low bone density [4], and low physical fitness [5]. Furthermore, children who are not physically active are denied the positive social and emotional benefits of physical activity including higher self esteem, lower anxiety, and lower stress [6]. A comprehensive understanding of the determinants of physical activity among youth is essential for the identification of appropriate points of intervention to promote active lifestyles and their associated health benefits. In this paper, we examine environmental influences on children's physical activity. Specifically, we review research assessing the association between attributes of the physical environment and children and adolescents' physical activity.

The physical or built environment has come to the forefront of public health research in the past 5 years, leading to a surge of research on environmental attributes and their associations with physical activity behaviors. A number of reviews have examined links between the physical environment and adults' physical activity [7-12]. Much less emphasis has been placed on research specific to children. One cannot assume that associations between the physical environment and physical activity among adults are applicable to children. As highlighted by Krizek, Birnbaum & Levinson [13], children in contrast to adults, spend large parts of their day at school, have considerable time for recreation, are more likely to accumulate physical activity through play, are not able to drive, and are subject to restrictions placed on them by adults.

Two reviews to date are specific to children. In 2000, Sallis et al. [14] published a comprehensive review of predictors of physical activity among youth. Studies published between 1970 and 1998 were included in the review. While this review does not focus on the physical environment, a small proportion of the 108 studies reviewed are specific to the physical environment. More recently, in 2005, McMillan [15] reviewed studies in both planning and public health literatures on urban form and children's trip to school. McMillan outlines policies and programs that may promote walking and cycling to school (e.g., Safe Routes to School) and highlights the lack of focus on children in the transportation literature. In the absence of research on environmental factors that affect children's trips to school, most of the studies reviewed by McMillan focus on adult populations.

In this descriptive review, we build on the work of Sallis et al. and McMillan by reviewing recent studies (published between 1990 and 2006) that examine the association between children's physical activity and environmental attributes (perceived and objectively measured). In particular, we provide specific information on the sample characteristics and design of each study, evaluate consistencies and inconsistencies in the literature, and identify gaps in the current research and possible avenues for future research. In addition, we broaden the set of children's behaviors from their trip to school as outlined by McMillan to physical activity in general. In order to serve both the need for understanding the link the physical environment and physical activity among youth and the implementation of next steps based on these findings, we use an organizing schema that identifies the parties responsible for specific elements in the built environment.

## Methods

### Definition of the physical environment

The physical environment is defined herein as objective and perceived characteristics of the physical context in which children spend their time (e.g., home, neighborhood, school) including aspects of urban design (e.g., presence and structure of sidewalks), traffic density and speed, distance to and design of venues for physical activity (e.g., playgrounds, parks and school yards), crime, safety and weather conditions. While crime and safety are not explicitly characteristics of the physical environment, they are included in this review as both are intimately linked with multiple characteristics of the physical environment including for example lighting, the condition of buildings, and the presence of trash. They also have vicinity effects such that a particular area can gain a reputation for safety or criminal activity.

### Identification of studies

Computer searches using PubMed, PsychInfo, EBSCO, CINAHL, and TRANSPORT were conducted in the English-language literature to identify published studies and reports examining relationships between the physical environment and children and adolescents' physical activity. Transportation and urban planning reports were accessed using TRANSPORT and general internet searches and by searching the bibliographies of papers. Search terms included physical activity, exercise, recreation, sport, walk/walking, cycle/cycling, transport, active commuting, environment, environmental determinants, physical environment, built environment, perceived environment, design, urban design, context, facilities, neighborhood, park, playground, situational factors, safety, crime and weather. These search terms are a compilation of the terms used in previous reviews [7-9,11,12]. In addition, the search terms children, child, adolescent, adolescence, youth, family, and parent were added to the terms list to limit studies to children and adolescents. The bibliographies of the identified studies were also reviewed for additional references.

Studies were not further considered if they (a) did not measure or model the environment (perceived or objective) and physical activity behaviors, (b) were descriptive in nature (e.g. qualitative studies were not reviewed), (c) only used a composite score of the environment that combined a number of environmental attributes and (d) did not report findings for children separately from those for adults. The database searches resulted in a total of 106 "hits", of which 29 were relevant and were further considered. Of the 29 studies, 23 met the selection criteria. Additional studies were identified through searching the bibliographies of articles. A total of 33 articles were identified for inclusion in the review.

### Recording and synthesizing research findings

For each study, the following were recorded: (a) first author and year; (b) sample characteristics such as sample size, gender, and ethnic/racial group of the participants; (c) age of participants; (d) research design, including whether it was cross sectional or longitudinal and whether perceived and/or objective environmental attributes were assessed; (e) the environmental variables that were examined; (f) the type of physical activity behavior assessed and the method of assessment; and (g) a summary of the significant associations that were identified (see Table 1 for a summary). This information was recorded by both authors and a graduate student and was cross checked to identify any inconsistencies. In instances in which multiple aspects of the physical environment were assessed, results for each component were recorded. If multiple measures of physical activity were included in the study, only results for the most comprehensive measure were recorded. For example, if a study included both objectively measured physical activity and a generalized self-report measure, results for the objective measure are reported. This simplification was necessary given the breadth of measures of physical activity used across studies. Simplifying the presentation of results for physical activity also served to maintain the focus of this review on the environmental correlates of physical activity broadly construed.

**Table 1:** Characteristics and main findings of the studies reviewed

**First Author (year)^ref#^**	**Number/Gender/Ethnicity/Country**	**Age group**	**Design**	**Environmental attributes (independent variables)**	**Physical activity behavior (outcome variable)**	**Significant associations with outcome variable**
Adkins (2004)^16^	52 F B USA	8- to 10-years	CS, P	There are playgrounds, parks and gyms nearby, it is safe to play outside (parent and child report)	Objectively measured (accelerometer) physical activity	No associations were identified between environmental attributes and physical activity.
Baranowski (1993)^41^	191 M/F B/W/H USA	3 and 4 years	CS, O	Month of the year (weather)	Directly observed physical activity.	Children were least active outdoors during the hottest months.
Boarnet (2005)^39^	62 M/F W/B/H/A USA	Parents of children in 3^rd ^– 5^th ^grade (8–10 years)	CS, O	Installation of sidewalks, crossing signals, traffic control as part of a Safe Routes to School (SR2S) program.	Parents' perceived change in child walking/biking to school	Greater increases in perceived rates of children walking/riding to school for children who passed a completed SR2S zone compared to those who did not pass a zone.
Braza (2004)^30^	105 students from 34 schools M/F W/B/H/A USA	5^th ^grade (ages 9 to 11 years) Unit of analysis: schools	CS, O	School size; population density; number of intersections per street mile in .5 mile buffer around school site. Data were obtained using Geographic Information Systems.	Rates of walking and biking to school among students surveyed in each school	Higher population density and a greater number of intersections per street mile were associated with higher rates of walking and biking to school in bivariate models.
Brodersen (2005)^17^	4320 M/F W/B/A England	11 to 12 years	CS, O	Area deprivation; number of sport pitches in borough; public spending on leisure facilities and open spaces; weather conditions.	Self-reported days during past week child performed hard exercise that made him/her breathe heavily and sweat.	Area deprivation (F) and total rainfall (F) were associated with lower physical activity. Colder temperatures (M) and number of sport pitches (F) were associated with higher physical activity.
Burdette (2005)^48^	3141 M/F W/B/H USA	3 years old	CS, P	Mothers' ratings of perceived neighborhood safety	Mothers' reports of the average time per day their child played outdoors	No associations between mothers' perception of neighborhood safety and their reports of the time their child spent playing outdoors
Carver (2005)^18^	347 M/F U Australia	12–13 years	CS, P	Parents' perceptions of good sports facilities for child, safe for child to walk/ride, good places for child to be active, traffic makes it difficult to walk. Child perceptions of ease to get around by bike, safety while walking/riding, roads safe, unattended dogs, strangers, fast food and convenience stores near home.	Child self-reported frequency and duration of walking or cycling in the neighborhood (for recreation, transport, exercise, get to school).	Adolescents walked or cycled more frequently when there were fewer unattended dogs (M, F), there were good places to be active (F), traffic was less problematic (M, F), there was lower perceived ease to cycle (M), there were more sport facilities in the area (M), the roads were perceived as safe(F), and convenience stores were further from home (F). Above is a simplified summary of results given number of variables assessed and analyses performed (i.e., >400 associations assessed).
Cohen (2006)^38^	1554 F W/H/B/A USA	6^th ^grade 12–13 years	CS, O	Distance to school along the shortest street network	Objectively measured (accelerometer) physical activity	A shorter distance to school was associated with greater MVPA during weekdays but not during the weekend
Dunton (2003)^19^	87 G W/H/A USA	14–17 years	CS, P	Perceived activity-related equipment in the home and activity-related resources in the community (e.g., park, gym, biking trail)	Self-reported vigorous physical activity, total energy expenditure, and leisure time activity	No associations were identified between activity-related resources in the home or the community and girls' self-reported physical activity.
Ewing (2004)^33^	726 people and 709 school trips surveyed U (gender) U(ethnicity) USA	Students K-12^th ^grade	CS, O	Estimated walk/bike time between destinations; proportion of street miles with street trees, bike lanes or paved shoulders, or sidewalks; sidewalk width; accessibility of attractions; neighborhood population density; school size	Likelihood of walking or biking to school	Students with shorter walk or bike times to school, and students traveling through areas with sidewalks on main roads were more likely to walk or bike to school. School size was not related to the likelihood of walking/biking to school.
Fein (2004)^20^	610 M/F W Canada	Grades 9–12 Mean age 15.5 years	CS, P	Home environment; convenient facilities (park, bike trails, gym, skating rink); School environment (gym space, availability of exercise equipment, athletic facilities accessible). The perceived importance of each resource was also assessed.	Self-reported physical activity	Perceived convenience of facilities and perceived home, neighborhood, and school environment were significantly correlated with self-reported physical activity. The perceived importance of each of these constructs was also associated with higher physical activity.
Felton (2002)^42^	1668 F W/B USA	8th grade (approx age 13 years)	CS, O	Urban/rural residence	Self-reported moderate and vigorous physical activity	White girls living in urban areas and black girls in rural areas reported higher vigorous activity than their respective counterparts.
Gomez (2004)^21^	177 M, F H USA	7th grade (approx age 12 years)	CS, P, O	Crime density (O); perceived neighborhood safety (P); distance to nearest play areas (O)	Self-reported participation in outdoor activities (not in school)	Greater proximity to play areas (M), lower crime density (F), and high perceived safety (F) were associated with higher outdoor activity.
Gordon-Larsen (2000)^43^	17766 M, F W/B/H/A USA	7^th ^to 12^th ^grade (approx ages 12 – 17 years)	CS, O	Urban/rural residence; crime; month of the year; region (South West, Midwest, Northeast).	Self-reported moderate to vigorous physical activity	Lower reported crime was associated with higher moderate to vigorous activity.
Hume (2005)^34^	127 M, F U Australia	10 year olds	CS, P	Children drew maps of their home and neighborhood environments. The frequency with which particular objects and locations were represented was coded including green space and outdoor areas and opportunities for physical activity in the neighborhood (e.g., playgrounds and facilities).	Objectively measured (accelerometer) physical activity.	Girls who drew a greater number of opportunities for physical activity in their neighborhood (e.g., the availability gyms, recreation and swimming centers, playgrounds) exhibited higher physical activity (specifically, low intensity physical activity).
Jago (2005)^40^	210 M W/B/H USA	10–14 years	CS, O	Ease of walking/cycling; tidiness of neighborhood; sidewalk characteristics; street access and conditions	Objectively measured (accelerometer) physical activity.	Sidewalk characteristics that foster walking (e.g., distance to curb, presence of trees as a buffer) were positively associated with light-intensity physical activity.
Molnar (2004)^44^	1378 M/F W/B/H USA	11 to 16 years	CS, P, O	Residents' perceived neighborhood safety and opportunities for children to play (P); social and physical disorder (O).	Hours/week participated in recreational physical activity (parent report).	More safe areas for children to play and lower social and physical disorder were associated with higher recreational activity.
Mota (2005)^32^	1123 M/F U Portugal	7^th ^– 12^th ^grade Mean age: 14.6 ± 1.6	CS, P	Adolescent reports of the activity-friendliness of their neighborhood (e.g. access to destinations, connectivity of streets, infrastructure for walking and cycling, neighborhood safety, aesthetics, and recreational facilities).	Self-reported physical activity	In comparison to low active adolescents, high active adolescents reported greater access to destinations such as stores and transit stops, higher neighborhood aesthetics, and more recreational facilities in their neighborhood.
Norman (2006)^37^	799 M/F W/H/B/A USA	11–15 years	CS, O	Number of private recreational facilities, schools and parks within 1 mile of home; walkability as assessed by residential density, retail floor area, intersection density, and land use mix	Objectively measured (accelerometer) physical activity.	Significant bivariate associations were found between moderate-to-vigorous PA and the number of recreation facilities (girls), the number of parks and measures of walkability including intersection density (girls), and retail floor area ratio (boys).
Sallis (1993)^22^	347 M/F W/H USA	4 years old	CS, P, O	Number of specified play spaces (e.g., friend's backyard, park) within walking distance of home (P); equipment at home (O).	Directly observed physical activity.	A greater number of specified play spaces within walking distance of home was associated with higher physical activity.
Sallis (1999)^45^	732 M/F W/A/PI/H USA	4^th ^– 5^th ^grade (ages 9 to 10 years) at baseline	L (20 months), P	Neighborhood safety (parent report)	Parent and child report of child physical activity. Objectively measured (accelerometer) physical activity	No links were identified between neighborhood safety and baseline physical activity or change in activity.
Sallis (2001)^23^	151 areas in 24 middle schools USA	Middle-school-aged students (approx ages 11 to 13 years)	CS, O	Type of play area (court space, open field space, indoor activity space); area size; permanent activity structures (e.g., basketball hoops, tennis courts, soccer goals); equipment.	Directly observed physical activity of students in each play area.	Higher levels of activity were noted when equipment was available in outdoor play areas (F) when more permanent activity structures were available (M), and when such structures were available in combination with adult supervision (F).
Sallis (2002)^28^	781 M/F W (75%) USA	Grades 1–12 (ages 6–18)	CS, P	Safe to play outdoors; access to parks/playgrounds; distance to park; safety of nearest park.	Parents' reports of children's physical activity and objectively measured (accelerometer) physical activity (N = sub sample of 200)	Among girls in grades 10–12, parents' perception of neighborhood safety was associated with higher physical activity. Among girls in grades 7–9, parents' perception of park safety was negatively associated with children's physical activity.
Sirard (2005)^47^	Unit of analysis = school (N = 8) USA	Elementary schools	CS, O	School urbanization and weather conditions.	Rates of walking and cycling to school for each school.	No associations between environmental variables and active commuting were identified.
Stratton (2005)^29^	99 M/F U Wales and England	4–11 years	I, O	Intervention in which school playgrounds were painted with murals, hopscotch, fun trails, snakes and ladders, and court markings (e.g., lines for basketball).	Heart rate telemeters were used to assess heart rate during physical activity and converted to represent MVPA and VPA.	In comparison to control schools, time spent in MVPA and VPA increased significantly in intervention schools as a result of playground painting.
Stucky-Ropp (1993)^31^	240 M/F W USA	5^th ^and 6^th ^grade Mean age: 11.2 ± .7	CS, P	Number of exercise-related items at home	Self reported physical activity	A greater number of exercise-related items in the home was associated with higher physical activity among girls but not boys.
Tappe (1989)^46^	236 M/F W, B, A USA	High school Mean age: 15 years 9 months	CS, P	Unsuitable weather as a barrier to exercise	Self-reported physical activity.	No differences in weather as a perceived barrier for physical activity among low and high active girls and boys.
Timperio (2004)^24^	1200 M/F U Australia	5–6 years and 10–12 years.	CS, P	Traffic density, road safety, strangers, sporting facilities, and public transportation (parent report). Children 10–12 years also reported on perceived traffic, road safety, strangers, and sport facilities.	Walking/riding to particular destinations (e.g., friend's house, park, school) 3 or more times/week (parent report)	Among 5–6 year olds, parents' perception of heavy traffic (M), and limited public transportation (F) were associated with lower walking/cycling among children. Among 10–12 year olds, youth who perceived no parks nearby (M, F) and whose parents believed that they had to cross many roads to get to play areas (M, F), that there were no lights or crossings (M), that there were few sporting arenas (F), and that there was limited public transportation (F) were less likely to bicycle/walk.
Timperio (2006)^36^†	912 M/F U Australia	5–6 years and 10–12 years	CS, O	Distance to school, busy-road barrier, route along busy road, pedestrian route directness (connectivity), steep incline	Walking or riding to school (parent report)	In both age groups, children were less likely to actively commute to school if their route as >800 m and a busy route barrier was present en route. Children with a steep incline (5–6 year olds) and a direct route to school (10–12 year olds) were less likely to actively commute
Trost (1997)^25^	202 (rural) M/F B/W USA	5^th ^– 6^th ^grade (ages 10 to 11) at baseline	L (1 year), P	Availability of activity-related equipment in the home.	Self-reported physical activity measured one year after determinants.	No links between home equipment and physical activity.
Trost (1999)^26^	108 M/F B USA	6^th ^grade (approx age 11 years)	CS, P	Availability of activity-related equipment in the home.	Objectively measured (accelerometer) physical activity	No links between home equipment and physical activity
Zakarian (1994)^27^	1634 M/F H/W/A/B USA	9^th ^and 11^th ^grade (approx age 14 and 16 years)	CS, P	Number of facilities for sport and exercise; safe to exercise in neighborhood.	Self-reported vigorous exercise (20 minutes of activity that makes your heart rate and breathing increase)	Access to facilities was associated with higher vigorous exercise.
Zask (2001)^35^	3912 M/F U Australia	5–12 years	CS, O	Direct observation of the availability of activity-related equipment (e.g., balls, fixed equipment)	Direct observation of children's physical activity behavior in all school playground areas.	The presence of equipment (other than balls) was not associated with children's physical activity.

Note:

Number/Gender/Ethnicity/Country: M, male; F, female; W, White; B = African American/Black; H, Hispanic/Mexican American; A, Asian; PI, Pacific Island; U = unknown (not mentioned); USA, United States of America.

Design: CS, cross sectional; L, longitudinal; I, intervention; P, perceived environment; O, objectively measured environment.

Physical activity behavior: MVPA, moderate to vigorous physical activity; VPA, vigorous physical activity.

Significant associations: M, F, significant findings limited to males and females respectively.

If an ethnic group made up ≤ 2% of the total sample, it was not included in the list of ethnic groups assessed.

† Given that the same sample was used in Timperio (2006) and Timperio (2004) and there was an overlap in the measure of physical activity, only the novel findings are recorded for the more recent study.

Findings from the studies are reviewed and synthesized using three a priori categories of environmental attributes including: (1) recreational infrastructure (e.g., the availability of parks/playgrounds, equipment in the home); (2) transport infrastructure (e.g., traffic speed/density, presence of sidewalks); and (3) local conditions (e.g., safety, crime, weather). These categories were chosen to facilitate the identification of the parties responsible for changing an environmental attribute and consequently possible avenues for intervention. Using a system similar to previous reviews on physical activity [7,14] it was noted whether a finding was positive and significant (+), negative and significant (-) or not statistically significant (0). This information is summarized in Table 2. In order to facilitate the comparison of findings across studies, results from bivariate (in contrast to multivariate) analyses are recorded and, where possible, results from bivariate analyses controlling for basic demographic variables (i.e., SES) are presented. Results are recorded separately for perceived and objectively measured attributes of the environment. Results specific to the perceived environment were further separated according to children's and adults' (usually parents) reports of the environment. The narrative review below accompanies the data presented in Table 2. Definitions and examples of each domain are provided, the responsible parties for each are identified, the key findings for each domain are summarized, and consistencies and inconsistencies are highlighted and possible explanations are provided.

**Table 2:** Pattern of findings for links between environmental attributes (perceived and objective) and children's physical activity.

	**Associations with physical activity**		
	
**Environmental Attribute**	**Perceived Environment**		**Objectively measured environment**
	Adult report	Child report	
**Recreational infrastructure**			
			
Private			
Home equipment		**0**^(19) ^**0**^(25) ^**0**^(26) ^**+**^(F)(31) ^**+**^(20)^	**0**^(22)^
Public			
Proximity of playgrounds and parks	**0**^(B)(16) ^**0**^(28) ^**+**^(22)^	**0**^(B)(16) ^**+**^(24)^	**+**^(M)(21)^
Availability recreation facilities	**0**^(28) ^**+**^(18)^	**0**^(F)(19) ^**+**^(27) ^**+**^(32) ^**+**^(20) ^**+**^(F)(24)^**+**^(F)(34)^	**+**^(F)(17) ^**+**^(F)(37)^
Spending on recreational infrastructure			**0**^(17)^
Distance to school (school location)			**-**^(36) ^**-**^(F)(38) ^**-**^(33)^
School size			**0**^(30) ^**0**^(33)^
Equipment/play structures in school play areas		+^(20)^	**0**^(35) ^**+**^(23) ^**+**^(29)^
**Transport infrastructure**			
			
Provision of amenities			
Presence of sidewalks		**0**^(32)^	**+**^(33) ^**+**^(39)^
Street and sidewalk conditions			**+**^(40)^
Presence of bike lanes/ease of cycling		**-**^(M)(18)^	**0**^(40) ^**0**^(33)^
Presence of controlled crossings	**+**^(M)(24)^		**+**^(39)^
Connectivity of street network		**0**^(32)^	**+**^(30) ^**-**^(36) ^**+**^(F)(37)^
Access to destinations		**+**^(32) ^**-**^(F)(18)^	**+**^(M)(37)^
Availability of public transportation	**+**^(F)(24)^		
Road hazards			
Number of roads to cross	**-**^(24)^		
Traffic (density/speed)	**-**^(M)(24) ^**-**^(18)^		**-**^(36)^
Pedestrian and cyclist safety	**+**^(F)(18)^		
Steep terrain			**-**^(36)^
**Local conditions**			
			
Safety and neighborhood disorder			
Perceived safety	**0**^(45) ^**0**^(B)(16) ^**0**^(28) ^**0**^(48) ^**+**^(44)^	**0**^(27) ^**0**^(B)(16) ^**0**^(32) ^**+**^(F)(21)^	
Area deprivation and crime			**-**^(F)(17) ^**-**^(F)(21) ^**-**^(43)^
Roaming dogs		**-**^(18)^	
Social disorder/stranger danger	**0**^(24)^		**-**^(44)^
Physical disorder/tidiness of area			**0**^(40) ^**-**^(44)^
Aesthetics of neighborhood		**+**^(32)^	
Region and weather			
Month of year (average temperature)			**0**^(43) ^**-**^11 ^**-**^(M)(17)^
Unsuitable weather		**0**^(46)^	**0**^(47) ^**-**^(17)^
Region of the United States			**0**^(43)^
Rural/suburban versus urban			**0**^(47)^**+**^(B)(42) ^**-**^(W)(42)^
Population density			**+**^(30) ^**0**^(33)^

Note:

^1 ^Associations identified with physical activity: +, significant positive association; -, significant negative association; 0, no association.

Superscript numbers = reference number.

Effects that are specific to particular demographic groups are noted as follows: M = males; F = females; B = Black/African American sample or subgroup

## Results

### Recreational infrastructure

Recreational infrastructure (play areas) for children can be classified as private (provided by their parents in and around the home), public (community areas or schools) or private-public (commercial play areas). Private recreational infrastructure may be subject to regulations associated with the property and issues of flexibility of use based on tenure of ownership (e.g., renter or home-owner). Public recreational infrastructure is primarily the responsibility of the municipality or agency charged with the provision of the original infrastructure, as is the maintenance and continued monitoring of the safety and condition of such assets. School yards, playgrounds and open space parks are most often considered public recreational infrastructure. Private-public recreational infrastructure includes youth camps, commercial clubs, and other businesses providing places for children to participate in physical activity. All of these recreational infrastructures are subject to land use regulations, including zoning codes. Twenty one studies that were reviewed examined the relationship between recreational infrastructure and children's physical activity [16-36]. The overwhelming majority (i.e., 19) of studies used a cross sectional design. One study used a 1-year longitudinal design and one used an intervention design. Five of the 21 studies used an objective measure of physical activity, including either accelerometry or heart rate monitoring, four used direct observation, 13 used a self-report measure, and one study used both objective and self-report measures. Seven studies used an objective measure of the environment (generally based on Geographic Information Systems), 12 used a self-report measure, and two studies used both methodologies. Finally, 12 of the studies were conducted in the US with the remaining studies being conducted in countries including Canada, England, Australia and Portugal.

#### Private recreational infrastructure

##### Home equipment

Four out of six studies found no association between home equipment and children's physical activity. Specifically, Sallis et al. [22] found no association between an objective assessment of equipment available in the home and observed levels of physical activity among preschool children. Dunton et al.[19] and Trost et al. [25] found no association between adolescents' reports of equipment in the home and their self-reported physical activity. A second study by Trost et al.[26] found no association between adolescents' reports of home equipment and their objectively measured physical activity using accelerometers. In contrast, Fein and colleagues [20] and Stucky-Ropp and DiLorenzo [31] found that the number pieces of exercise equipment in the home was positively and significantly associated with higher self-reported physical activity among adolescents girls and boys and young adolescent girls (but not boys).

Differences in the results outlined above cannot be explained by differences in sample size, participant age, or the operationalization of home equipment. Differences, however, may be explained by differences in the ethnic composition of the samples; both studies identifying a significant effect for home equipment used a predominantly white sample, whereas, studies that did not identify an effect used either an exclusively African American sample [26] or samples of mixed racial/ethnic background [3,19,22]. It should also be noted that both studies identifying a significant positive effect used a self-report measure of physical activity. The remaining studies used self-report [19,25] or an objective assessment [22,26] of physical activity. Thus, any association identified between home equipment and children's physical activity is limited to white adolescent samples and to self-report measures of physical activity.

#### Public recreational infrastructure

##### Proximity of parks and playgrounds

A significant positive association between the proximity of parks and playgrounds to the home and children's physical activity was identified in three out of five studies. In an exclusively Hispanic sample, Gomez et al[21] found that objectively measured distance to the nearest play area was inversely associated with adolescent boys', but not girls', self-reported physical activity. Sallis et al. [22] found that parents' reports of the number of play areas within walking distance of the home were positively associated with observed levels of physical activity among preschool children. Furthermore, Timperio, et al. [24] found that children who reported a lack of parks or sports grounds near their home made fewer walking and cycling trips. In contrast to these studies, Sallis et al. [28] and Adkins et al. [16] (using an exclusively Black sample) found no association between proximity of playgrounds and parks and children's objectively measured physical activity.

Although a number of ethnic/racial groups were assessed across studies, no consistent ethnic/racial differences were identified. Differences in methods used to assess physical activity, however, were noted for studies that did and did not identify a significant association. Both studies that found no association [16,28] assessed physical activity using accelerometers, which provide an aggregate measure of physical activity across a number of days. In contrast, studies that found a significant association relied on self-reported or observed physical activity, both of which are prone to reporter/observer bias, but which can be tailored to provide a specific measure of physical activity (e.g., walking or cycling trips).

##### Availability of recreation areas and spending on recreational infrastructure

In eight out of ten studies, a significant positive association was identified between the availability of recreation areas, or the presence of such areas in the vicinity of the home, and children's physical activity. Among Australian samples, Timperio et al. [24] found that parents' reports of few sporting arenas in the area were linked with lower rates of walking and cycling among girls and Carver et al. [18] found that parents' reports of the presence of good sporting facilities nearby for their children were associated with higher self-reported walking or cycling among adolescent girls and boys (A simplified summary of the results from Carver et. al are presented throughout this review given the extensive number of variables assessed. Only results for the frequency of walking/cycling in general are reported). In a study combining qualitative and quantitative methods, Hume et al. [34] found that, when children were instructed to draw pictures of their home and their neighborhood, girls who drew more opportunities for physical activity, including recreational facilities such as gyms and swimming centers, had higher objectively measured physical activity. Among US samples, Zakarian et al. [27] found that a greater number of facilities for sport and exercise in the area (based on self report) were associated with higher adolescent self-reported vigorous activity and Brodersen et al. [17] found that the number of sport pitches in the borough, as determined by objective assessment, was associated with higher self-reported vigorous activity among girls but not boys. Similarly, Norman et. al. [37] found that objective measures of the number of recreational facilities and parks within a mile of the home were associated with higher objectively measured physical activity among adolescent girls, but not boys. Finally, Mota et al. [32] and Fein et al.[20] using samples from Portugal and Canada respectively, found that adolescents' reports of the availability of facilities such as swimming pools, playgrounds and parks were associated with higher self-reported physical activity. In contrast to the aforementioned studies, Dunton et al. [19] found no association between girls' reports of activity-related resources in the community and their self-reported physical activity and Sallis et al. [28] found no association between access to facilities and children's objectively measured physical activity. In addition, no association was identified between spending on recreational infrastructure and children's self-reported physical activity [17].

With one exception, there are no obvious differences in the designs of studies that did and did not identify a significant association between the availability of recreational areas and children's physical activity. Specifically, there were no clear differences across studies in the definition of recreational facilities (which usually included structures such as swimming pools, gyms, sporting arenas, and parks), the methods used to assess physical activity, or the demographic characteristics of the samples. There were, however, clear differences is sample size across studies. The majority of studies that identified a significant effect used samples of 1000 or more participants. In contrast, the two studies that found no effect used samples of approximately 100 participants, taking age and gender break-downs into consideration. This suggests that the association between the availability of facilities and physical activity among youth is relatively small and therefore only measurable with a large sample. While the availability of facilities was assessed in all studies, no studies directly asked children or parents whether they used such facilities. Consequently, the association between recreational facilities and physical activity is indirect at best.

##### School characteristics

Three out of three studies identified a negative association between distance to school and children's physical activity. Timperio et al.[36] and Cohen et.al. [38] (girls only) found significant negative associations between an objective measure of distance to school and children and adolescents' objectively measured moderate to vigorous physical activity. Ewing et al. [33] found that lower walk/cycle time to school, an indirect measure of distance, was associated with higher rates of active commuting to school. In contrast to studies assessing distance to school, Braza et al. [30] and Ewing et al. [33] found no association between school size, an indirect measure of whether or the school is located in a residential area and therefore close to homes, and the rates of walking and cycling to school.

With respect to characteristics within schools, Sallis et al. [23] found that middle-school-aged children were more likely to be active during school recess periods when there was a larger number of activity-related equipment (e.g., balls) and the permanent activity structures (e.g., basketball hoops) available; these effects were most notable in the presence of adult supervision. Similarly, Fein et al. [20] found that adolescents' reports of the availability of sports equipment, the functionability of equipment, and access to athletic facilities at school were associated with higher self-reported physical activity. In contrast, Zask et al. [35] found no association between the availability of playground equipment (with the exception of balls) and children's physical activity. Finally, in an intervention examining the effect of playground markings such as hopscotch and court lines for basketball on children's physical activity, Stratton and Mullan [29] found significant increases in moderate to vigorous physical activity and vigorous physical activity in intervention schools relative to control schools.

In sum, three out of three studies found that children who live close to schools are more likely to actively commute to school and three out of four studies found that children were more active during play periods when characteristics of school play areas (e.g., access to equipment, permanent play structures, and marked courts) facilitated physical activity. No associations, however, were found between school size and children's physical activity. The lack of effects of school size reported by Braza et al. [30] and Ewing et al. [33] may be attributable to the use of aggregate data, or data collected at one level (e.g., a census track) that is then aggregated to a higher level (e.g., county). As a result of the process of aggregation, any information pertaining to individual residences or specific locations is lost.

### Transport infrastructure

Two types of transport infrastructure were examined in studies including the provision of amenities (e.g., sidewalks, crossings) and the presence of road hazards. Transportation infrastructure in urban areas is the responsibility of a number of agencies. For example, in the United States, Metropolitan Planning Organization (MPO) are generally charged with the preparation of planning documents and the allocation of funding for major programs and projects, whereas, the designation of crosswalks, traffic signals, pedestrian signage, and other amenities are in general the responsibility of various transportation departments based on right-of-way and public ownership of property. Nine studies assessed associations between transport infrastructure and children's physical activity [18,24,30,32,33,36,37,39,40]. All nine studies used a cross sectional design. Two studies used an objective measure of physical activity and six studies used an objective measure of the environment. The remaining studies relied on self-report instruments. Five of the nine studies were conducted in the US; the remaining studies were conducted in Australia and Portugal.

#### Provision of amenities

##### Presence and condition of sidewalks and bike lanes

Results generally supported a positive association between the presence and condition of sidewalks and children's physical activity with three out of four studies identifying a significant positive effect. Ewing et al. [33] found that the proportion of street miles with sidewalks was positively associated with children's rates of walking or cycling to school. In an evaluation of the implementation of a Safe Routes to School program, Boarnet et al. [39] found that children who passed areas in which sidewalks were installed were more likely to walk or cycle to school than children who did not pass such areas. In contrast, Mota et al. [32] found no association between the perceived presence of sidewalks on streets in the neighborhood and adolescents' self-reported activity. In the only study that assessed the impact of sidewalk conditions, Jago and colleagues [40] found that objectively assessed sidewalk characteristics such as the distance from the sidewalk to the curb, average height of trees, and sidewalk material and type were associated with higher objectively measured light intensity physical activity (e.g., slow walking) among children. The studies that identified significant effects used objective measures of the environment and measured children's walking (or low intensity physical activity) as the outcome variable, which is the most likely component of physical to be influenced by sidewalk characteristics. In the only study that failed to identify a significant effect, a self report measure of sidewalk availability was used along with a generalized measure of physical activity that may not reflect subtle differences in physical activity that result from the presence of sidewalks.

With respect to infrastructure for cycling, Jago et al. [40] found no association between the ease of cycling (presence of bike lanes, attractiveness for cycling, number of read lanes) and objectively measured light intensity physical activity in a sample of boys and Ewing et al. [33] found no association between the presence of bike lanes and children's walking/cycling to school. Furthermore, Carver et al. [18] found that the perceived ease of cycling was associated with lower (rather than higher) rates of cycling among boys. Spurious findings for the presence of bike lanes or ease of cycling may be explained by a number of factors including the use of a measure of physical activity that cannot detect cycling (i.e., accelerometers) [40], low rates of bicycling to school in general [33], and inflated type II error due to performing an extensive number of analyses [18].

##### Presence of controlled crossings, street connectivity, and access to destinations

Two studies examined the association between the presence of controlled crossings (e.g., presence of lights, crossings, or crosswalks) and children's physical activity, both of which identified significant positive effects. Timperio et al. [24] found that parents' reports of a lack of traffic lights and controlled crossings were associated with lower rates of walking and cycling among boys, but not girls. In their evaluation of a Safe Routes to School program, Boarnet, et al. [39] found that children who passed areas in which traffic control methods were installed were more likely to walk or cycle to school than children who did not pass such areas.

Conflicting results were found for studies assessing street connectivity with only two out of four studies identifying a significant effect in the anticipated direction. Braza et al. [30] found that an objective measure of street connectivity was associated with higher rates of walking or biking to school. Similarly, Norman et al.[37] found that higher intersection density (also assessed using an objective measure) was associated with higher objectively measured moderate-to-vigorous physical activity among girls but not boys. Mota et al. [32], however, found no associations between perceived street connectivity and adolescents' self-reported activity. In contrast to what might be expected, Timperio et al. [36] found that a more direct route to school (i.e., higher connectivity, which was assessed using objective methods), was associated with lower rates of walking and cycling to school among older children (10–12 years); no links were found between connectivity and active commuting to school among younger children (5–6 years of age).

The difference in findings reported by Mota et al. versus Braza et al. and Norman et al. may reflect the possibility that effects of connectivity are only observed when objective measures of connectivity are used; it is possible that individuals are not able to accurately recall and report the level of street connectivity in their neighborhood. The findings outlined by Timperio et al., which were opposite to those expected (with higher connectivity or a more direct route associated with lower rates of active commuting to school), are more difficult to explain. Timperio et al. suggest that the counterintuitive effects of connectivity in their study may reflect the possibility that children's travel behavior is more influenced by traffic safety concerns than street networks.

Three out of four studies identified a significant positive association between access to destinations and children's physical activity. This consistent pattern was noted although a variety of measures of access were used across studies including the presence of destinations such as shops, access to public transportation, and retail floor area ratio (i.e., ratio of retail building square footage to parcel square footage). Timperio et al. [24] found that parents' reports of a lack of public transportation were associated with lower rates of walking and cycling among girls but not boys. Mota et al. [32] found that the ability to walk to destinations such as shops and transit stops was associated with higher physical activity among adolescents and Norman et. al.[37] found that a greater retail floor area ratio (reflecting greater retail space and access to shops) was associated with higher objectively measured moderate to vigorous physical activity among adolescent boys but not girls. In contrast to expectations, Carver et al. [18] found that adolescent girls' reports of greater access to convenience stores reported lower, rather than higher, rates of walking for transport. The general consistency of results for access to destinations, despite differences in its operationalization, suggests that it should be considered further in future investigations.

#### Road hazards

A variety of road hazards have been examined across studies including the number of roads to cross, the presence of a road barrier, traffic speed and density, pedestrian and cyclist safety, and terrain. All three studies assessing road hazards found a negative association between such hazards and children's physical activity. Timperio et al. [24] found that parents' reports that their children had to cross many roads to get to a play area (girls and boys) and of high levels of traffic density in their local area (boys only) were associated with lower rates of walking and cycling among children. In a second study by Timperio et. al. [36], using the same sample but using an objective assessment of the environment, the presence of a busy road barrier (e.g., a highway) en route to school (5–6 years olds and 10–12 year olds) and the presence of a steep incline (5–6 year olds only) were associated with lower rates of active commuting to school. Similarly, Carver et al. [18] found that parents' reports of traffic impeding the ability to walk were associated with lower rates of walking or cycling among girls and boys, whereas, parents' perception of the roads in the area being safe was associated with a higher frequency of walking among girls (but not boys). It is worth noting that all of these studies were conducted with urban Australian samples.

### Local conditions

Both recreational and transport infrastructures exist within the context of local community conditions. The actions of other community members and agencies such as police patrols, community clean-up programs, and/or transient populations, all exert influence at the local level. These conditions include both positive and negative environmental attributes such as general neighborhood safety, safety of play areas, crime rates, social disorder and stranger danger, physical disorder and weather conditions. Eighteen studies were identified that assessed links between local conditions and children's physical activity [16-18,21,24,27,28,30,32,40-48]. All but one study used a cross sectional design. Four studies used an objective measure of physical activity (accelerometry), one used direct observation, and fifteen studies relied on a self-report measure of physical activity. With regard to measures of the environment, nine studies used a self-report measure, seven studies used an objective measure and two studies used both methods. The vast majority of studies (13 out of 18) were conducted in the US.

#### Safety and neighborhood disorder

##### Safety, crime, and area deprivation

Nine studies examined the association between perceived safety and children's physical activity. These studies overwhelming reported a null effect with seven [16,27,28,32,45,48] of the nine studies showing no association between perceived safety and children's physical activity. The lack of an association was not limited to a particular research design or sample population. Two exceptions to the pattern of null findings are the studies by Molnar, et al. [44] and Gomez et al. [21]. In Molnar et al. residents' reports of the safety of children's local play areas were positively associated with parents' reports of their children's participation in recreational physical activity. Similarly, Gomez et al. [21] noted that adolescents' reports of perceived neighborhood safety were associated with higher self-reported outdoor physical activity for girls but not boys. The general lack of findings for perceived safety may reflect the fact that most of the studies measured general levels of physical activity, which may or may not be linked with neighborhood safety given that children can be active outside their neighborhood.

In contrast to perceived safety, three out of three studies identified a significant negative association between crime or area deprivation and children's physical activity. Gordon-Larsen et al. [43] and Gomez et al. [21] (girls only) found significant inverse associations between objectively measured crime rates and adolescents' self-reported physical activity. Similarly, Brodersen et al. [17] found that area deprivation (i.e., rates of car ownership, housing tenure, unemployment and overcrowding in the district) was associated with lower self-reported physical activity among 11–12 year old girls but not boys. Finally, Carver et al. [18] found that the presence of roaming dogs were associated with lower rates of walking or cycling among adolescents.

##### Social and physical disorder and neighborhood aesthetics

Three studies assessed links between neighborhood disorder and children's physical activity. Findings were mixed across these studies, likely reflecting differences in the operationalization of disorder. Molnar [44] objectively measured physical (e.g., graffiti, empty beer bottles) and social (e.g., alcohol in public, people selling drugs) disorder using coded video recordings and direct observation of neighborhoods. Both forms of disorder were associated with lower levels of parent-reported recreational activity among adolescents. Jago et al. [40], however, found no association between an objective measure of neighborhood tidiness and children's objectively measured physical activity. Likewise, Timperio et al. [24] found no association between children's perceptions of stranger danger (a source of social disorder) and parents' reports of their walking and cycling to destinations. Thus, it appears that any association between neighborhood disorder and physical activity may be limited to much higher levels of disorder (or deviance) such as those measured by Molnar et al. A general lack of tidiness or the perception that strangers can be dangerous but may not be enough to dissuade youth from being active outdoors. In the only study that assessed perceived aesthetics, Mota et al. [32] found that adolescents' reports of the aesthetics of their neighborhoods (i.e., there are many interesting things to look at while walking) were positively associated with their self-reported physical activity.

#### Region and weather

##### Weather

A significant association between weather and children's physical activity was identified in two out of five studies. Baranowski et al. [41] and Brodersen et al. [17] found that preschool children and 11–12-year-old boys respectively were less active during hotter months of the year. Brodersen et al. [17] also found that higher rainfall was associated with lower self-reported physical activity among girls but not boys. Although "unsuitable" weather was reported by adolescents as a perceived barrier to physical activity in the study by Tappe et al. [46], such perceptions were not associated with lower levels of self-reported physical activity. Similarly, Gordon-Larsen et al. [43] found no relationship between the month of the year and adolescents' self-reported physical activity, indicating a lack of a seasonality effect. Finally, Sirard et al. [47] found no association between weather conditions and rates of walking and biking to school.

The effects of weather may have been underestimated in these studies due to the restricted time range in which the data were collected. For example, Gordon-Larsen et al. [43] used data collected on physical activity between April and December. It is possible that the effect of bad or unsuitable weather was eliminated by the exclusion of the months of January through March (winter months in the northern hemisphere where the research was conducted). There was also limited variability in the geographic region within each study. No studies collected data across multiple regions that varied in the suitability of the climate for outdoor activity. Consequently, inconsistent or non-significant effects could be explained by a general lack of variability in the data by month of the year and/or location. Furthermore, no studies considered the availability of resources for indoor recreational activity in communities. It is likely the unsuitable weather conditions will most often be associated with low levels of physical activity in communities in which there are few opportunities for indoor physical activity.

##### Region, urban/rural location, and population density

Three studies examined associations between region and children's physical activity, with one of the three studies showing a significant effect. Gordon-Larsen et al. [43] found that residence in the Northeast of the United States was associated with higher self-reported physical activity among adolescents in comparison to residence in the South, West or Midwest. This effect of region could be explained by a myriad of factors such as regional differences in weather, income, education, ethnic/racial make-up, and access to community resources. When examining rural/suburban versus urban location, Sirard et al. [47] found no differences in rates of walking and biking to school for schools located in urban and suburban areas. Felton et al. [42] found mixed results for location. White girls in urban areas were more active (based on self-reports) than White girls in rural areas. The opposite was found for Black girls; black girls living in rural areas were more active than Black girls from urban areas. Although the difference was not discussed by the authors, it is possible that White girls from urban areas lived in neighborhoods in which they could take advantage of the infrastructure for physical activity generally attributed to urban areas such as the presence of sidewalks and accessible parks. While Black girls may also have had access to similar resources, their ability to use such resources may have been limited by neighborhood characteristics such as crime.

Two studies assessed links between population density and children's active commuting to school; no consistent effects were identified. Ewing et al. [33] and found no association between population density in the immediate area around children's homes and their rates of walking/cycling to school. In contrast, Braza et al. [30] found that higher population density was associated with higher rates of active commuting to school. Neither study considered whether children attended their local school, rather than a magnet or private school outside of the local area, or the feasibility of children walking or riding to school.

## Discussion

In this paper we reviewed research on associations between the physical environment and children's physical activity while highlighting the parties responsible for each environmental attribute. This was achieved by classifying and reviewing studies specific to recreational infrastructure, transport infrastructure and local conditions. The most consistent pattern of findings was evident for transport infrastructure, followed by recreational infrastructure, with the least consistent pattern of results noted for local conditions. Although there were no consistent differences in results across age or ethnic groups, there was some indication that associations between environmental characteristics were more commonly noted for girls than boys.

### Summary of findings

Results from studies examining components of transport infrastructure showed that children were more active when there were sidewalks in their neighborhood, they had destinations to walk to, public transportation was available, there were fewer uncontrolled intersections to cross, and traffic density was low. Results were more consistent for the absence of roads hazards (i.e., roads to cross, traffic density/speed) than the provision of amenities (i.e., sidewalks, presence of destinations, controlled intersections). In addition, findings were most consistent for parents' reports of infrastructure followed by objective measures; in general, null findings, or findings in the opposite direction to those anticipated, were evident for studies relying on children's reports of transport infrastructure. No consistent differences by gender or ethnic group emerged for transport infrastructure.

Although findings were less consistent for recreational infrastructure, there were a number of instances in which the majority of studies supported a particular relationship. The majority of studies showed that the availability of facilities in neighborhoods and the availability of equipment and permanent activity structures in school play areas were associated with higher physical activity. In addition, greater distances to school were associated with lower rates of walking and cycling to school. In contrast to expectations, most studies failed to identify an association between home equipment and children's physical activity and results for the proximity to playgrounds were mixed. Some gender differences in the reported associations were apparent. Six out of seven effects specific to girls were significant and in the anticipated direction. Most of these effects were noted for child reports of the environment. In contrast, only one significant effect was specific to boys. In two instances, associations specific to African Americans were reported. In both cases, no significant effects of recreational infrastructure were present for this demographic group.

Findings were least consistent for local conditions, reflecting the broader range of characteristics assessed. In general, no effects were found for perceived neighborhood safety or the perceived safety of play areas. However, both studies that used objective measures of crime rates reported a significant negative association between crime and children's physical activity. Similarly, objectively measured area deprivation and the perceived presence of roaming dogs were associated with lower physical activity. No consistent pattern of findings was evident for region or weather conditions. With respect to differences noted by gender, three effects specific to girls were significant and in the anticipated direction; only one effect was specific to boys and this was also in the anticipated direction. Of the three effects specific to African Americans, two were not significant and one was in the opposite direction to that expected.

### Recommendations for future research

The research reviewed herein generally reflects the first "phase" of research on links between the physical environment and children's physical activity. In this first round of research, many of the methodologies were in a developmental stage. As a result, there was little consistency in the methods used. In addition, in many cases, the methods used and the designs adopted were opportunistic as researchers grappled with which research questions to pose. Greater rigor with regard to measurement of both physical activity and the environment, and the use of more sophisticated designs will facilitate the establishment of a transdisciplinary approach, which is imperative to moving this body of research into the "next phase".

#### Measurement issues

The ability to measure characteristics of the physical environment is greatly facilitated by the use of geographic information systems (GIS). Of the 33 studies reviewed, 6 used GIS-based methodologies. As the use of GIS becomes more commonplace, it is imperative that the methods for "creating" and displaying the data are recorded in detail (the "meta data" – the data about the data). Currently, there is little description of the various processing decisions that are made when using GIS in published research. The absence of such information slows research progress and inhibits the comparison of findings across studies and research disciplines. While we advocate for the incorporation of GIS into research designs, the perceived environment should also be taken into consideration because people's perceptions may, in fact, motivate their behavior more than the true nature of the situation.

In contrast to the objective assessment of the environment, objective measures of physical activity were more widely incorporated into the studies reviewed with 13 out of 33 studies reviewed using an objective measure of physical activity (8 used accelerometers, 4 used direct observation, 1 used heat rate monitoring). Using accelerometers to measure children's physical activity and/or directly observed children's activity removes the possibility of response bias, particularly among children [49]. Although the use of objective measures of physical activity is preferable, because it allows greater confidence in the validity of the assessment, objective measures may not be feasible in large-scale survey research due to financial and logistical constraints. In addition, accelerometers provide only a generalized measure of physical activity and do not provide information on the type of activity or the location in which physical activity takes place. As is noted by Giles-Corti et al. [50], assessing context-specific behaviors is key to understanding associations between the physical environment and physical activity. In many of the studies reviewed, generalized measures of physical activity were implemented that may not be sensitive to specific environmental attributes. Consequently, null effects may reflect a lack of specificity in the measures used rather than the absence of an association. New equipment that incorporates Global Positioning Systems (GPS) into accelerometers may facilitate the ability to obtain context-specific measures of physical activity by making it possible to know exactly where (i.e., longitude/latitude data) and when (i.e., electronically time-stamped data) the physical activity occurred.

#### Design issues

With two exceptions, all studies relied on cross sectional analysis. Given the need to understand behavioral changes associated with environmental attributes, longitudinal studies are more appropriate. Such studies will help us determine whether the pattern of results reflects the ability of the environment to constrain or facilitate certain behaviors or reflects the type of person/family who chooses to live in certain neighborhoods [9,51]. In addition to using a longitudinal design, more complex models need to be developed and tested. With the exception of gender, research has rarely examined factors that may moderate the link between the environment and children's physical activity (i.e., interact with the environment to predict physical activity). The use of simplistic designs with little consideration of moderating factors as highlighted by McMillan [15], may lead to simplistic and erroneous conclusions. The most noteworthy example is the general failure to consider ethnicity, family income, or neighborhood deprivation as possible moderating or confounding variables. Furthermore, many studies have assessed children across a broad age range, which ignores the possibility that associations between the physical environment and physical activity may be age-specific due to differences in parental control and children's independent mobility.

In addition to a lack of emphasis on children's age, the role that parents play in regulating children's use of the physical environment has not been considered in research to date. Consequently, the assumption is generally that there is a direct link between the environment and children's physical activity. This is unlikely to be the case given children's lack of decision autonomy and the role that parents play as gate keepers to children's use and exploration of the physical environment surrounding their home. Research shows that parents' decisions about their children's independent mobility are influenced by a number of factors such as their perceptions of the safety of the area, neighborhood relations, and proximity to a park [52]. Research designs and techniques will need to link quantitative and qualitative data to successfully understand the nature of parents' decision-making processes and their willingness to allow their children to participate in physical activity under a combination of environmental attributes.

#### The need for a transdisciplinary approach

Scientists from different research paradigms have largely approached research of children and physical activity from the perspective of their own discipline with little integration of ideas and methods across disciplines. To most effectively assess the impact of the physical environment on physical activity levels among youth, future research will need to adopt a transdisciplinary approach that draws upon diverse research disciplines such as geography and planning, public health, exercise science, criminal justice and human development. Such an approach will require greater standardization of procedures and detailed reporting of these procedures than has generally been exhibited in research to date. In addition, a transdisciplinary approach will require clear communication and dialogue across research disciplines, including access to literatures across various research communities. In many cases, information on environmental attributes is contained in planning documents rather than in refereed journals. These documents are often available over the Internet, but may not be included in databases or other researchable tools.

### Summary and conclusion

In this review, we have found preliminary evidence that a relationship exists between children's participation in physical activity and environmental attributes. Limitations of this review include the exclusion of studies not published in English or searchable in English-based databases, the overall bias against publishing studies with null results, and the lack of research specific to children outside the health sciences. Future work could enhance our understanding of this important topic by assessing both perceived and objective characteristics of the environment, including objective measures of children's physical activity and the physical environment, adopting longitudinal designs, assessing the interaction between various environmental attributes, and examining the important role that parents play as gate keepers to children's use of the physical environment. There is also a need for studies outside the US to determine whether results identified using US samples can be generalized internationally. Finally, we advocate the continued use of the classification scheme outlined herein as this will allow us to determine the parties responsible for attributes found to influence children's physical activity and to make the necessary changes. We also strongly recommend the establishment of a transdisciplinary research agenda sufficiently transparent to facilitate the sharing of information across a growing body of work being generated by diverse research approaches.
